# Corticosteroid-Induced Psychiatric Disorders: Mechanisms, Outcomes, and Clinical Implications

**DOI:** 10.3390/diseases12120300

**Published:** 2024-11-23

**Authors:** Lara Nasereddin, Omar Alnajjar, Homam Bashar, Sara Feras Abuarab, Rahma Al-Adwan, Dinesh Kumar Chellappan, Muna Barakat

**Affiliations:** 1 Department of Clinical Pharmacy and Therapeutics, Faculty of Pharmacy, Applied Science Private University, Amman 11937, Jordan; laranasereddin@gmail.com (L.N.);; 2 MEU Research Unit, Middle East University, Amman 11831, Jordan; 3 Department of Pharmaceutical Sciences and Pharmaceutics, Applied Science Private University, Amman 11937, Jordan; 4 Department of Life Sciences, School of Pharmacy, International Medical University, Bukit Jalil, Kuala Lumpur 57000, Malaysia

**Keywords:** corticosteroid-induced psychiatric disorders, HPA axis dysregulation, corticosteroids and mental health, psychosis and corticosteroids, corticosteroid side effects

## Abstract

Corticosteroids are extensively used in medicine for their powerful anti-inflammatory and immunosuppressive effects. However, their psychiatric side effects—such as mood disturbances, anxiety, and psychosis—are significant yet often underappreciated. This review provides a comprehensive exploration of corticosteroid-induced psychiatric disorders, with a focus on their underlying mechanisms and clinical implications. We examine how corticosteroids influence the hypothalamic–pituitary–adrenal (HPA) axis, leading to the dysregulation of stress responses and alterations in neurotransmitter levels, particularly dopamine, serotonin, and glutamate. These changes are linked to structural abnormalities in key brain areas such as the hippocampus and amygdala, which are implicated in mood and anxiety disorders, psychosis, and conditions like post-traumatic stress disorder (PTSD) and eating disorders. This review highlights the need for healthcare providers to be vigilant in recognizing and managing corticosteroid-induced psychiatric symptoms, especially in vulnerable populations with pre-existing mental health conditions. The complex relationship between corticosteroid type, dose, duration, and mental health outcomes is explored, emphasizing the importance of personalized treatment approaches to mitigate psychiatric risks. Given the widespread use of corticosteroids, there is an urgent need for more focused research on their psychiatric side effects. This review underscores the importance of patient education and careful monitoring to ensure optimal therapeutic outcomes while minimizing mental health risks associated with corticosteroid therapy.

## 1. Introduction

Corticosteroids are a class of steroid hormones naturally produced by the adrenal glands, playing a crucial role in regulating inflammation, immune response, metabolism, and stress adaptation [1,2]. Over the past century, synthetic corticosteroids have been seen as “magic drugs” and have become a cornerstone in the treatment of a wide array of medical conditions, including autoimmune diseases, respiratory disorders, and inflammatory diseases [3]. Their widespread use is attributed to their potent anti-inflammatory and immunosuppressive properties [3]. However, the benefits of corticosteroid therapy come with significant side effects, which can vary depending on the dosage, duration, and type of corticosteroid administered [3,4,5].

While physical side effects such as weight gain, osteoporosis, and fluid retention are well known, there is growing evidence that corticosteroids also pose substantial risks to mental health [6]. Psychiatric complications, ranging from mood disturbances to severe psychiatric disorders such as depression, anxiety, mania, and even psychosis, are often under-recognized by both the general public and healthcare providers [3]. Symptoms of corticosteroid-induced psychiatric complications typically include hallucinations, delusions, and paranoia. A longitudinal cohort study reported that psychiatric disturbances were more common in patients receiving higher doses of corticosteroids, with 18.6% of patients on more than 80 mg of prednisolone or methylprednisolone experiencing psychiatric symptoms, compared to 1.3% of patients on lower doses [7]. These symptoms usually appear within 3–4 days of starting corticosteroid therapy and often resolve within a week of discontinuation. These findings primarily reflect the effects of acute treatment. However, prolonged corticosteroid exposure can also pose significant psychiatric risks, with different symptom profiles emerging over time [8]. The correlated neuropsychiatric effects are believed to arise from the disruption of the hypothalamic–pituitary–adrenal (HPA) axis, a central stress response system that regulates cortisol levels in response to stress [9]. Prolonged corticosteroid exposure can lead to dysregulation of this system, contributing to a range of mental health disorders [10,11,12].

Long-term use of corticosteroids has been associated with higher incidences of depression, anxiety, and cognitive decline. For example, a cohort study by Fardet et al. (2013) found that patients on long-term glucocorticoid therapy had a significantly elevated risk of psychiatric disorders, including severe neuropsychiatric outcomes, compared to those on short-term regimens [7]. Another study reported that chronic corticosteroid use is linked to increased depressive symptoms, with rates of cognitive dysfunction more pronounced in older adults [10].

Further evidence highlights that both dose and duration play critical roles in determining psychiatric outcomes. Higher concentrations (e.g., >40 mg/day) are more likely to trigger manic and psychotic symptoms, especially in acute scenarios, while lower chronic doses can lead to persistent mood disturbances and anxiety over time [10]. Therefore, a nuanced approach is required when evaluating the psychiatric risks of corticosteroids, considering both the concentration and duration of exposure.

Despite the increasing awareness of corticosteroid-induced psychiatric symptoms, the exact mechanisms remain poorly understood, and the clinical guidelines for managing these side effects are limited. This narrative review aims to explore the role of cortisol and synthetic corticosteroids in psychiatric diseases, highlighting the neurobiological pathways involved and offering critical insights for healthcare providers [13]. By reviewing the existing literature, this paper seeks to enhance awareness of the psychiatric risks associated with corticosteroid use and emphasize the need for tailored treatment approaches when prescribing these medications, particularly in patients with pre-existing or susceptible mental health conditions.

## 2. Methodology

The review aimed to identify, synthesize, and critically evaluate the existing literature addressing corticosteroid-induced neuropsychiatric outcomes, including their impact on the hypothalamic–pituitary–adrenal (HPA) axis and brain function.

### 2.1. Data Sources

A comprehensive literature search was carried out using databases such as PubMed, Scopus, and Google Scholar. The search was limited to studies published between 2000 and 2024, using the following keywords: corticosteroids, glucocorticoids, depression, anxiety, psychosis, cognitive changes, HPA axis, mental health disorders, and mood disorders.

### 2.2. Study Selection Process

A total of 450 articles were initially identified through the database search (the PRISMA flow diagram is provided in the Supplementary Materials). After removing duplicates (90 articles), 360 articles were screened based on titles and abstracts. Articles were excluded if they did not meet the inclusion criteria. Of these, 175 articles were excluded for not focusing on corticosteroid-induced mental health disorders, HPA axis effects, or relevant neuropsychiatric conditions. Full-text reviews were conducted on 185 articles, and 59 articles were excluded due to irrelevance to neuropsychiatric outcomes or corticosteroid use. Ultimately, 126 articles were included in this review.

### 2.3. Inclusion Criteria

Articles included in the review had to meet the following criteria: published in peer-reviewed journals, in English; focused on corticosteroid-induced mental health disorders (e.g., depression, anxiety, psychosis, cognitive dysfunction); discussed the physiological effects of corticosteroids or glucocorticoids on the HPA axis and brain function; and human studies or animal studies were included only if directly relevant to human clinical conditions.

### 2.4. Exclusion Criteria

Studies were excluded if they were not peer-reviewed, not published in English, did not focus on non-psychiatric side effects of corticosteroids (e.g., metabolic effects), did not directly investigate neuropsychiatric outcomes or HPA axis dysfunction, or were animal studies lacking clear relevance to human mental health.

### 2.5. Data Extraction and Synthesis

Relevant data from the selected studies were extracted, including study objectives, methods, key findings, and conclusions. The studies were categorized based on the psychiatric disorders examined (e.g., depression, anxiety, bipolar disorder, psychosis) and the key mechanisms explored, such as HPA axis disruption, glucocorticoid receptor interactions, and corticosteroid-induced changes in brain function. Additionally, articles discussing the long-term cognitive and mood impacts of corticosteroid therapy were highlighted.

### 2.6. Limitations

This review is subject to potential publication bias and the inherent variability in study designs and methodologies across the included articles. A significant portion of the studies were observational, which may introduce confounding factors and limit the ability to infer causality. Future systematic reviews and meta-analyses are recommended to provide a more quantitative assessment of the relationship between corticosteroid use and mental health outcomes.

## 3. Mental Health Disorders and Corticosteroids

### 3.1. Depression

Depression is a highly prevalent mental health disorder and one of the leading causes of disability globally, affecting over 280 million people, including children and adolescents [14,15]. Its symptoms include persistent sadness, loss of interest in previously enjoyable activities, and feelings of worthlessness, which may lead to suicidal ideation [15,16,17]. A variety of factors contribute to the development of depression, including genetic predisposition, environmental stressors, and physiological changes [18].

**Figure 1:**
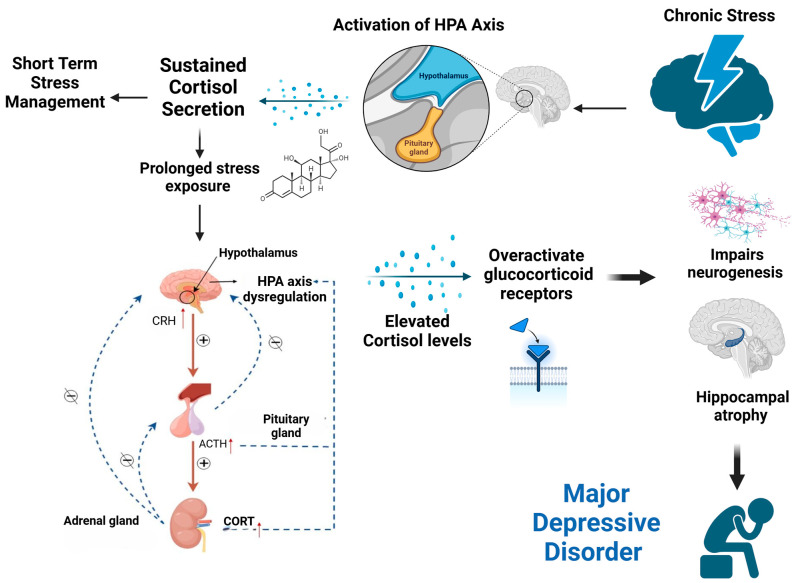
Chronic stress-induced HPA axis dysregulation leads to elevated cortisol, impaired neurogenesis, hippocampal atrophy, and the development of major depressive disorder.

One key physiological contributor to depression is the HPA axis, as illustrated in Figure 1. Chronic stress initiates the activation of the HPA axis, involving the hypothalamus, which releases corticotropin-releasing hormone (CRH). CRH stimulates the pituitary gland to release adrenocorticotropic hormone (ACTH), which in turn prompts the adrenal glands to secrete cortisol—a hormone vital for managing acute stress [19].

However, with prolonged exposure to stress, sustained cortisol release disrupts the HPA axis’s normal feedback mechanisms, resulting in HPA axis dysregulation [19]. In this dysregulated state, the usual negative feedback loop fails, meaning that high cortisol levels no longer effectively signal the hypothalamus and pituitary gland to reduce CRH and ACTH production. This persistent activation leads to chronically elevated cortisol levels, which overactivate glucocorticoid receptors (GRs) in the brain, particularly within the hippocampus [18,20].

The overactivation of GRs has significant consequences for brain function and structure. It impairs neurogenesis (the formation of new neurons) and causes hippocampal atrophy (a reduction in hippocampal volume), both of which are strongly linked to the development of major depressive disorder (MDD) [21,22]. Research has shown that chronic exposure to elevated glucocorticoid levels not only affects brain plasticity but also contributes to changes in cognition, behavior, and emotional regulation, particularly in older adults [23]. Studies in animals have demonstrated that prolonged glucocorticoid treatment induces hippocampal neuron death, further supporting the link between HPA axis dysregulation and depressive-like behavior [23]. In other words, increased glucocorticoid levels in the hippocampus are believed to contribute to changes in cognition, dementia, and depression in older adult individuals [23]. They directly influence brain architecture and behavior by binding to specific receptors in the central nervous system.

Glucocorticoids contribute to the development of depression independently of stress performance [24]. An experiment has shown that rats exposed to chronic stress do not exhibit depressive behavior in the absence of glucocorticoid production. Despite reduced levels of the neurotransmitter serotonin (5-HT), which plays a key role in various psychiatric disorders, including major depression, the intended effects remain a topic of investigation [25]. Studies have shown that chronic corticosteroid use, particularly synthetic glucocorticoids like prednisone, mimics the effects of prolonged stress, exacerbating depression symptoms in vulnerable individuals [26,27,28]. This is particularly evident in patients with Cushing’s syndrome, where endogenous cortisol overproduction is linked to high rates of depression [29]. In experimental models, prolonged exposure to glucocorticoids has been shown to induce depressive-like behaviors, providing further evidence of the connection between HPA axis dysregulation and depression [30,31].

Furthermore, using inhaled corticosteroids seems to partially inhibit asthma-induced depression. Asthma is an allergic airway disease, and a very common chronic health condition associated with a high likelihood of developing depression. This association may be attributed to the nature of the disease, which can lead individuals to endure challenging experiences such as difficulty sleeping or exercising. Additionally, individuals may become dependent on an inhaler for their well-being [32,33]. Inhaled corticosteroids are the most common anti-inflammatory agents for asthma treatment and are an essential step in controlling the condition based on asthma guidelines. Studies also showed that inhaled corticosteroids reduce brain volume alteration and other structural changes associated with asthma patients [34,35,36]; this suggests the utilization of inhaled corticosteroids such as fluticasone and budesonide may have protective effects on allergic asthma-induced depression, in addition to their core role in managing airway inflammation [37].

Corticosteroids can have an effect on depression symptoms depending on the amount and form of administration [38]. In general, higher doses and systemic administration methods (such as oral or intravenous delivery) are more likely to cause mood changes, including symptoms of depression. In contrast, inhaled corticosteroids, commonly used for asthma, have lower systemic absorption, which may explain their relatively lesser impact on mood compared to oral corticosteroids [39,40].

Corticosteroids are supplied differently and have different effects on the body, which can have a variety of consequences for mental health. Furthermore, the duration of medication, individual vulnerability, and the presence of pre-existing mood disorders can all influence how patients experience depressive symptoms when taking corticosteroids [38].

Despite the clear association between corticosteroids and depressive symptoms, managing corticosteroid-induced depression remains challenging. While antidepressants can be prescribed, their efficacy in the context of glucocorticoid-induced depression is variable [39,40]. Additionally, the discontinuation of corticosteroids may alleviate symptoms, but this is not always feasible, especially for patients requiring long-term treatment [21]. Therefore, healthcare providers must remain vigilant when prescribing corticosteroids, particularly for patients with a history of depression or those exhibiting early signs of mood disturbances. Early intervention and careful monitoring of psychiatric symptoms are essential to prevent the escalation of corticosteroid-induced depression.

### 3.2. Bipolar Disorder

Bipolar disorder (BD) is a chronic mental illness characterized by extreme fluctuations in mood, energy, and behavior, making it challenging for individuals to carry out daily activities [41]. Patients experience alternating episodes of depression (a down phase marked by sadness and lack of energy) and mania or hypomania (an up phase characterized by elevated mood, hyperactivity, and impulsive behavior). Some patients may even exhibit mixed episodes, where symptoms of both mania and depression occur simultaneously [41].

BD is categorized into several subtypes, with bipolar I and bipolar II being the most common. In bipolar I, manic episodes are more severe and often require hospitalization, whereas in bipolar II, manic episodes (hypomania) are less intense but more difficult to treat [42]. Regardless of the subtype, mood episodes in BD typically occur several times per year, with depressive symptoms often being more prominent than manic symptoms [42]. This disorder carries a high burden of morbidity and mortality. It is estimated that over 40 million individuals worldwide are affected by BD, with an average onset age of 25 years [13]. Genetics plays a significant role in the etiology of BD, as 80–90% of those diagnosed with the condition have a family history of either bipolar disorder or depression [43]. Additionally, patients with BD often face significant social and functional impairments, including withdrawal from social relationships, decreased productivity, and an increased risk of suicide—the highest rate among psychiatric disorders [44,45].

Diagnosing BD is often delayed, as symptoms are frequently misattributed to other psychiatric conditions. To establish a diagnosis, a patient must exhibit manic symptoms for at least seven consecutive days or require hospitalization [46]. In addition, specific criteria for depressive episodes must be met.

One of the challenges in diagnosing and managing BD is the interaction between the disorder and certain medical treatments, particularly corticosteroids. Corticosteroids, especially when used in high doses or for prolonged periods, are known to induce manic or hypomanic episodes in susceptible individuals. Patients with autoimmune diseases, who commonly require corticosteroid therapy, are at particular risk [47]. Elevated cortisol levels, whether endogenous (as in Cushing’s syndrome) or exogenous (as with corticosteroid use), have been linked to the emergence or exacerbation of mood disorders, including BD [48]. For example, a woman diagnosed with BD experienced multiple hospitalizations due to what was later identified as Cushing’s disease. Once her cortisol levels were normalized, her psychiatric symptoms significantly improved [48]. In parallel, children with asthma, who are frequently treated with inhaled corticosteroids, have also been linked to an increased risk of developing BD [49]. Additionally, corticosteroid-induced secondary mania is particularly concerning in elderly populations, as they are more susceptible due to the frequent use of medications and comorbid neurological conditions [50]. Even low doses of corticosteroids have been reported to induce manic symptoms in patients without prior psychiatric histories [51].

Environmental factors, such as stress, sleep disruption, and substance use, are known triggers for mood episodes in vulnerable individuals. Although the exact pathophysiology of BD remains unclear, imbalances in brain chemicals, including dopamine and serotonin, are believed to play a role in the dysregulation of mood [45]. Corticosteroids, due to their influence on the HPA axis and glucocorticoid receptors, are considered potent triggers of manic episodes in patients with BD [45]. Several case reports have highlighted the profound impact of corticosteroids on BD. For instance, one patient who was on high doses of corticosteroids (dexamethasone and prednisolone) for breast cancer treatment experienced acute manic symptoms despite being on maintenance therapy for BD [52]. Another case involved a woman who developed mania after being prescribed prednisone for sinusitis, with her symptoms resolving after the discontinuation of the corticosteroid [53].

There are proposed interactions between corticosteroids and neurotransmitters that could contribute to symptoms of bipolar disorder or affect mood regulation. Corticosteroids can influence several neurotransmitter systems which include serotonin, dopamine, norepinephrine, and glutamate [54]. Corticosteroids may reduce serotonin levels or receptor sensitivity, which can lead to mood disturbances [54]. Elevated levels of cortisol (a primary corticosteroid) can affect dopamine pathways, potentially exacerbating mood swings or manic symptoms [54]. Corticosteroids can influence norepinephrine levels, which are associated with arousal and mood regulation, potentially leading to depressive or manic episodes [54]. Some studies suggest that corticosteroids can increase glutamate levels, which may impact excitability and mood [54]. The interaction of these neurotransmitters is complex, and the effects can vary among individuals. For those with a predisposition to mood disorders, corticosteroids may trigger or worsen symptoms, contributing to the cycling seen in bipolar disorder. Moreover, stress responses, which are influenced by corticosteroids, can also play a role in mood dysregulation [54].

Currently, there is no standardized treatment for managing steroid-induced bipolar disorder (S-IB) [55]. However, the use of mood stabilizers and antipsychotics has shown promise in treating corticosteroid-induced mania [56]. In contrast, combining antidepressants with mood stabilizers appears to offer limited benefit in this context [57]. Early identification and intervention, along with careful monitoring of corticosteroid use in patients with BD, are crucial to minimizing the risk of mood destabilization.

### 3.3. Mania

Mania is a severe mental state characterized by a noticeable and often disruptive change in an individual’s usual behavior, lasting for a minimum of one week. This shift typically results in significant impairment in social, occupational, or personal functioning [58]. Mania is most often associated with BD-I, though it can also appear in other conditions, including those triggered by external factors like corticosteroid use [58]. Symptoms of mania may include heightened talkativeness, rapid speech, a reduced need for sleep, difficulty concentrating, and increased physical and mental activity. These symptoms can lead to impulsive or reckless behavior, often necessitating hospitalization [58].

The hallmark symptoms of mania are summarized by the mnemonic “DIG FAST”: distractibility, irresponsibility (or irritability), grandiosity, flight of ideas, increased activity, decreased sleep, and excessive talkativeness [58]. This pattern of symptoms can vary in intensity but is typically characterized by a significant departure from the individual’s baseline mood and behavior. The pathophysiology of mania is closely linked to the neurobiological mechanisms underlying BD [59]. Research suggests that hyperactivity in the amygdala and reduced activity in the prefrontal cortex are critical contributors to the impairment of executive functions observed in manic patients [60]. This imbalance in brain activity, particularly the overactivation of regions involved in emotional regulation, may explain the impulsive and erratic behaviors characteristic of manic episodes.

On the other hand, secondary mania can be triggered by pharmacological agents like corticosteroids, is often more challenging to manage than primary bipolar mania. It is especially common in elderly patients, who are at higher risk due to the use of chronic medications and the presence of comorbid neurological diseases [50]. Several cases of steroid-induced mania have been reported, even with low doses of corticosteroids, highlighting the potent psychiatric effects these drugs can have [50]. Additionally, mania is relatively common, with studies reporting a lifetime prevalence of approximately 4% in the general population [60]. Among individuals aged 18–24, the prevalence of manic episodes is about 7.5%, with a higher risk of developing comorbid conditions such as anxiety disorders, substance abuse, and suicidality [61]. Interestingly, up to 80% of monozygotic twins may develop mania if one twin has been diagnosed with BD, indicating a strong genetic component, although the lack of a 100% concordance rate suggests that environmental factors, such as corticosteroid use, also play a role [58].

Corticosteroid consumption has been linked to manic symptoms via a variety of hypothesized pathways [62]. One important aspect is neurotransmitter imbalance, namely increased dopamine activity, which has been related to manic symptoms. Furthermore, prolonged cortisol increase caused by corticosteroid treatment can disrupt the hypothalamic–pituitary–adrenal (HPA) axis, contributing to mood instability [62]. Changes in inflammatory indicators can impact mood and precipitate manic episodes, therefore inflammation plays a role as well [62]. Because sleep and mood stability are so closely linked, corticosteroid-induced sleep disruptions can worsen mood dysregulation. Individuals with a hereditary predisposition to mood disorders may be more prone to these effects, as corticosteroids can impair cognitive processes and increase irritability and impulsivity, all of which are associated with mania [62]. These elements, when combined, can provide a favorable setting for the onset of manic symptoms in susceptible individuals [62].

Early identification of corticosteroid-induced mania is crucial for timely intervention. This may involve tapering off the corticosteroid dosage or, in cases where discontinuation is not feasible, introducing mood stabilizers or antipsychotics to control the manic symptoms. Preventative strategies, particularly for high-risk individuals, include careful monitoring and patient education regarding the potential psychiatric side effects of corticosteroid therapy.

### 3.4. Schizophrenia

Schizophrenia is a severe mental disorder characterized by symptoms that distort an individual’s perception of reality, including delusions, hallucinations, and negative symptoms such as emotional flatness and reduced social engagement [63,64]. To be diagnosed with schizophrenia, these symptoms must persist for at least six months, leading to significant social, occupational, or interpersonal disruptions [63,64,65]. Cognitive deficits, a core feature of schizophrenia, further complicate its treatment, as they affect memory, attention, and executive functioning [66,67].

Several environmental factors, including childhood adversity, urban living, and chronic stress, are known to increase the risk of developing schizophrenia [68]. These factors may trigger or exacerbate underlying vulnerabilities, contributing to the disorder’s complex etiology. Schizophrenia affects approximately 1 in 300 people globally, with a significantly reduced life expectancy—between 10 and 20 years less than that of the general population—largely due to high suicide rates and comorbid physical illnesses such as cardiovascular and metabolic disorders [63,69]. Although the exact cause of schizophrenia remains unknown, increasing evidence points to stress-related pathophysiology involving the HPA axis. This stress response system regulates cortisol levels in the body, and when continuously activated by chronic stress, it may lead to HPA axis hyperactivity. Elevated cortisol levels, in turn, impact dopaminergic neurotransmission, reducing dopamine in the mesocortical neurons while increasing dopaminergic activity in the mesolimbic system [70,71,72]. These fluctuations are believed to contribute to the hallmark negative and cognitive symptoms of schizophrenia, supporting the “dopaminergic hypothesis” of the disorder [72].

Corticosteroids, which mimic the effects of cortisol, have been implicated in psychiatric disturbances, including psychosis. Continuous exposure to high cortisol or corticosteroid levels may trigger or worsen psychosis in susceptible individuals [63,71]. This is particularly relevant in schizophrenia, where elevated cortisol levels can further exacerbate symptoms. Additionally, studies have shown an increase in pituitary volume among individuals with schizophrenia, further linking HPA axis dysfunction to the disorder [73,74].

Recently, the role of immune dysregulation in schizophrenia has garnered attention and several studies have linked it with autoimmune diseases, suggesting that individuals with schizophrenia are more prone to developing autoimmune conditions later in life [75,76,77]. Moreover, research has uncovered the presence of autoantibodies targeting neural structures, such as N-methyl-D-aspartate receptors (NMDARs) and neural cell adhesion molecules (NCAMs), which may contribute to the development of psychotic symptoms [78,79,80]. These findings highlight the potential autoimmune basis of schizophrenia and support further exploration of immune-based therapies.

Research into the relationship between schizophrenia and corticosteroid use has yielded mixed results [81,82,83]. While some studies have suggested that corticosteroid therapy may increase the risk of psychosis, others have found no statistically significant association between corticosteroid use and schizophrenia, particularly in asthma patients who frequently use inhaled corticosteroids [80]. One study compared individuals who received corticosteroid prescriptions with those who did not and found an increased risk of early diagnosis of schizophrenia and psychosis among corticosteroid users [84]. In parallel, a randomized, placebo-controlled trial investigated the effect of corticosteroids as an adjunct treatment for schizophrenia symptoms. In this study, individuals diagnosed with schizophrenia or related disorders were given prednisolone for six weeks alongside antipsychotic medication. The results showed no significant improvement in symptom severity compared to the placebo group [85]. This suggests that while corticosteroids may influence inflammatory pathways in the brain, their use in treating schizophrenia remains inconclusive and warrants further investigation.

Given the complex relationship between schizophrenia, cortisol levels, and immune function, it is clear that corticosteroids play a multifaceted role in the disorder. Although their direct link to schizophrenia is still under debate [38], corticosteroids’ impact on the HPA axis and immune system suggests that they may exacerbate symptoms in vulnerable populations. Careful monitoring and judicious use of corticosteroids in patients with schizophrenia are essential to avoid triggering or worsening psychotic episodes.

### 3.5. Anxiety Disorders

Anxiety is one of the most prevalent mental health disorders worldwide, characterized by excessive worry and fear, often about future events or potential dangers [86]. This disorder affects multiple levels of functioning—emotional, cognitive, behavioral, and physiological. On an emotional and cognitive level, anxiety manifests as persistent apprehension and discomfort, often accompanied by distorted beliefs and pessimistic thinking. Behaviorally, anxious individuals may exhibit procrastination, avoidance, and difficulty concentrating [87]. Physiologically, the symptoms of anxiety are driven by increased sympathetic nervous system activity, leading to tachycardia, elevated blood pressure, sweating, and tremors [87,88].

The *Diagnostic and Statistical Manual of Mental Disorders* (DSM-5) outlines several types of anxiety disorders, including generalized anxiety disorder, social anxiety disorder, panic disorder, and separation anxiety disorder, among others. Each type of anxiety disorder is defined by specific diagnostic criteria, though all share the common characteristic of excessive fear and worry [87]. Risk factors for anxiety disorders are multifaceted and include genetic predisposition, environmental stressors, and childhood experiences such as shyness or distress when facing new situations [89]. Epigenetic changes caused by prolonged stress are believed to play a role in the dysregulation of the body’s stress response, contributing to the development of anxiety [90].

Many studies have investigated the neural underpinnings of anxiety, showing altered activation patterns in brain regions responsible for processing and regulating emotions, such as the amygdala and prefrontal cortex [90,91]. Neuroimaging studies, including functional MRI (fMRI), have further demonstrated that psychological therapies can help normalize these activation patterns, leading to symptom improvement in anxiety and related mental health disorders [92,93].

According to the WHO, around 4% of the global population experienced some form of anxiety in 2019 [94]. More recent studies suggest that anxiety is particularly prevalent in regions with higher levels of socioeconomic development and urbanization, and it is more commonly diagnosed in women than in men [95]. Similarly to the other psychiatric disorders, the hypothesis linking anxiety to corticosteroid use revolves around the HPA axis, which regulates the secretion of glucocorticoids, such as cortisol, during stressful situations [96]. Cortisol binds to GRs and mineralocorticoid receptors (MRs), both of which are located in brain regions responsible for managing fear and anxiety [97,98,99]. While MR activation helps to enhance alertness and mobilize energy in response to acute stress, GR activation triggers negative feedback mechanisms that eventually attenuate the stress response, preserving homeostasis [100]. However, prolonged or chronic exposure to stress can lead to an imbalance between GR and MR activation. This imbalance has been linked to structural changes in the brain, including atrophy in the hippocampus and increased dendritic branching in the amygdala, both of which contribute to HPA axis dysregulation [83]. This impaired ability to regulate stress is thought to play a role in the development of anxiety disorders [101,102].

Exogenous corticosteroids, which are synthetic analogs of endogenous glucocorticoids, are commonly used to treat various medical conditions such as asthma, allergies, and autoimmune diseases [97,101,103]. However, like endogenous cortisol, synthetic corticosteroids can affect the brain’s stress-regulating systems. Designed to have a stronger affinity for GRs, synthetic corticosteroids have been associated with an increased risk of anxiety disorders [104,105]. A meta-analysis published in 2023 found that approximately 8% of individuals using glucocorticoids developed anxiety disorders, further supporting the link between corticosteroid use and anxiety [106]. Similarly, a retrospective study conducted at King Abdulaziz Medical City found a 0.95% prevalence of anxiety, particularly among female patients, who were on long-term oral corticosteroid therapy, with prednisolone being the most frequently prescribed drug [38].

Therefore, local corticosteroids (e.g., inhaled corticosteroids) and systemic corticosteroids have been implicated in impairing executive cognitive functions and increasing the risk of mood and anxiety disorders in adults [105]. Interestingly, a 2023 animal study suggested that pre-treatment with inhaled corticosteroids might mitigate some brain changes associated with asthma, indicating a complex relationship between corticosteroids and anxiety [10]. While, for managing corticosteroid-induced anxiety, common medications used for chronic anxiety disorders include benzodiazepines (BZDs), selective serotonin reuptake inhibitors (SSRIs), tricyclic antidepressants (TCAs), and monoamine oxidase inhibitors (MAOIs) [37]. Although there is no specific protocol for prescribing corticosteroids to patients with pre-existing anxiety disorders, healthcare providers should exercise caution and carefully monitor patients for psychiatric symptoms [107].

### 3.6. Post-Traumatic Stress Disorder (PTSD)

Post-traumatic stress disorder (PTSD) is a complex mental health condition that arises following exposure to traumatic events. PTSD is characterized by the re-experiencing of trauma through intrusive memories, nightmares, or flashbacks, causing significant emotional distress [108]. Individuals with PTSD often display persistent negative alterations in mood and cognition, such as distorted blame, diminished interest in activities, and negative beliefs about oneself or others [108,109]. Behavioral symptoms include avoidance of trauma-related stimuli, emotional numbing, hypervigilance, and heightened arousal, which can manifest as irritability, concentration difficulties, and reckless behaviors [110].

The DSM-5 no longer classifies PTSD solely as a disorder of fear and anxiety, recognizing that it involves a broader spectrum of negative emotional responses and unique pathophysiological mechanisms [111,112]. Research on the neurobiology of PTSD highlights significant alterations in neurotransmitter and neurohormonal systems. Patients with PTSD often exhibit normal to low cortisol levels coupled with elevated levels of corticotropin-releasing factor (CRF), which contributes to an exaggerated stress response and heightened sympathetic arousal [111]. This dysregulation leads to increased norepinephrine release and hyperactivity in the anterior cingulate cortex, intensifying stress responses such as elevated heart rate and hyperarousal [113].

Additionally, PTSD is associated with decreased gamma-aminobutyric acid (GABA) activity and increased glutamate levels, both of which contribute to dissociative experiences and emotional dysregulation. Serotonin deficits in the brain’s emotion-regulating regions, such as the amygdala and hippocampus, further exacerbate the symptoms [114]. Structurally, individuals with PTSD often exhibit reduced hippocampal volume, heightened amygdala reactivity, and impaired functioning of the medial prefrontal cortex, underscoring the profound impact of PTSD on brain structure and function [113].

Given the role of corticosteroids in stress regulation, prolonged corticosteroid use can potentially exacerbate PTSD symptoms by further dysregulating the HPA axis and altering neurotransmitter balance. While corticosteroids may be essential for managing certain physical health conditions, their psychiatric side effects, particularly in patients vulnerable to PTSD, warrant careful monitoring and dosage management.

### 3.7. Eating Disorders (EDs)

Eating disorders, such as bulimia nervosa and anorexia nervosa (AN), are serious mental health conditions characterized by disordered eating behaviors [115]. Bulimia nervosa involves binge eating episodes followed by compensatory behaviors to prevent weight gain, such as excessive exercise or self-induced vomiting [115]. Anorexia nervosa, on the other hand, is marked by a persistent desire to lose weight, often through unhealthy methods like restrictive eating or over-exercising, despite being underweight [66,116]. Both conditions disproportionately affect females, particularly during adolescence, but males can also be affected [66,116].

The rising incidence of eating disorders in recent decades has been attributed to factors such as media influence, unrealistic body image standards, and societal changes in dietary habits [117]. Research suggests that eating disorders are multifactorial, involving interactions between genetic, behavioral, and psychological factors [118]. In individuals with anorexia nervosa, prolonged malnutrition leads to hyperactivation of the HPA axis, resulting in elevated cortisol levels as the body attempts to maintain glucose homeostasis during periods of low energy availability [119]. This state of hypercortisolemia may predispose individuals with AN to complications related to stress regulation, including the development of mood disturbances.

Corticosteroids, known to increase appetite and promote weight gain, can be particularly concerning for individuals with eating disorders [120]. Long-term corticosteroid use may exacerbate body image concerns and lead to psychological distress in individuals predisposed to eating disorders [120]. Although rare, cases of eating disorders emerging after corticosteroid therapy have been reported, likely linked to corticosteroid-induced mood disturbances, such as depression, which can amplify disordered eating behaviors [12,121]. It is essential for healthcare providers to educate patients about the potential side effects of corticosteroid therapy, including weight gain, to mitigate the psychological impact on individuals at risk for eating disorders.

### 3.8. Special Cases: Corticosteroid-Induced Psychosis During COVID-19

During the 2003 epidemic of SARS-CoV, systemic corticosteroids were given to patients who suffered from severe respiratory complications because of the infection. A meta-analysis was then performed to study the overall effect of corticosteroid use; it was found that corticosteroids resulted in a higher mortality rate. When another systematic review and meta-analysis were completed to measure the effect of corticosteroids on influenza, prolonged ICU stay, higher mortality rates, and secondary fungal and bacterial infections were also found [122,123]. In 2020, when the SARS-CoV-2 spread, the WHO interim guidance on the clinical management of severe acute respiratory infection announced an advisory not to use corticosteroids for these cases [124].

Regardless, the Infectious Diseases Society of America (IDSA) strongly recommends the use of 6 mg IV or PO dexamethasone or alternative corticosteroids until discharge in critically ill patients to treat acute respiratory distress syndrome. The level of recommendation decreases as the severity decreases [125]. Coronavirus disease 2019 (COVID-19) is an infection caused by the SARS-CoV-2 virus (severe acute respiratory syndrome coronavirus 2) and is associated with a vast array of symptoms. It frequently spreads among persons who come into close contact. It was first found in China in 2019 and became a pandemic in 2020. The most frequent symptoms are fever, chills, and a sore throat, but there are many others. Around 80% of cases experience mild symptoms [126,127,128]. However, some experience immune dysregulation, which produces acute respiratory distress syndrome (ARDS) and may lead to ICU admission [127]. Among the side effects of the infection, psychosis has been reported. The etiology of why psychosis happens is still unknown. Yet, it is predicted that inflammatory responses in the central nervous system (CNS) produced by COVID-19 and corticosteroids are the reason behind this phenomenon. Also, drugs used in the ICU can induce psychotic symptoms [86,128,129,130]. Multiple cases have been reported regarding this issue.

Psychiatric symptoms may appear 3–4 days after the initiation of corticosteroid treatment. Symptoms normally clear up a week after corticosteroid cessation. HPA axis suppression and variations in neurotransmitter levels are possible reasons behind corticosteroid-induced psychosis (e.g., elevated dopamine brain activity) [8]. Multiple cases have been reported after receiving corticosteroids; symptoms were relieved after corticosteroid discontinuation and antipsychotic initiation.

The first step in managing corticosteroid-induced psychosis is to taper off the dose of the corticosteroid or discontinue it. Despite that, this option alone is not possible for all patients [122]. Sometimes, it is best to keep the corticosteroid dose as it is or, when the symptoms are severe, it is best to start the patient on an adjuvant medicine for management [123]. It was reviewed that atypical (second-generation) antipsychotics are the first line in managing corticosteroid-induced psychosis [122], as they are considered to have a safer side effect profile when compared to other medications [122,123]. Antipsychotics should be used for a short period, with gradual discontinuation when the patient is in the remission state with no visible psychiatric symptoms [123].

Neuroleptics and TCAs should be avoided in cases of steroid-induced psychosis as they have the potential to exacerbate symptoms. However, recent studies have reported the beneficial results of using TCAs in the treatment of symptoms occurring due to corticosteroid withdrawal. Lithium prophylaxis has also been studied; however, it has been proven to improve mood episodes that happen along with psychotic disorders. Therefore, it is considered a mood stabilizer rather than an antipsychotic and should not be used in the treatment of steroid-induced psychosis as a first-line treatment. The vast majority of patients with steroid-induced psychosis recover from the condition within approximately two weeks [131].

### 3.9. Summary of the Key Mechanisms

**Figure 2:**
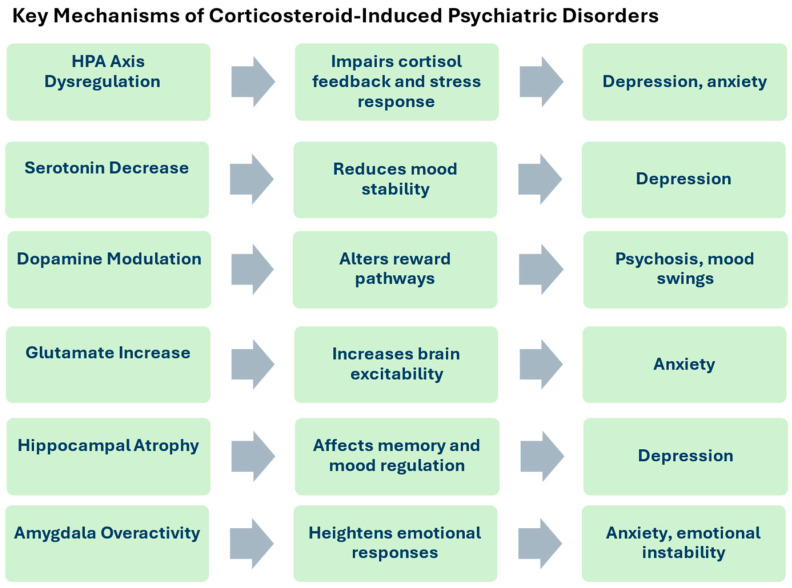
The key mechanism of corticosteroid-induced psychiatric disorders, through HPA axis dysregulation, neurotransmitter imbalances, and brain structure changes, contributing to depression, anxiety, and emotional instability.

In this review, it is proposed that the psychiatric effects of corticosteroids can be attributed to key neurobiological mechanisms, as shown in Figure 2. Prolonged corticosteroid exposure disrupts the HPA axis, impairing the body’s stress response and feedback regulation. This disruption, coupled with neurotransmitter imbalances, contributes to psychiatric symptoms. Specifically, corticosteroids reduce serotonin levels, leading to depression; modulate dopamine pathways, increasing the risk of psychosis and mood swings; and elevate glutamate levels, heightening anxiety. Furthermore, corticosteroids induce structural brain changes, such as hippocampal atrophy, associated with memory deficits and depression, and amygdala overactivation, contributing to anxiety and emotional instability. These mechanisms underscore the complexity of corticosteroid-induced psychiatric symptoms and highlight the importance of tailored monitoring and intervention strategies.

## 4. Conclusions

Corticosteroids are often hailed as “magic drugs” for their wide-ranging therapeutic applications, from treating inflammatory diseases to managing autoimmune conditions. However, their psychiatric side effects, including mood disturbances, anxiety, and psychosis, are increasingly recognized as significant concerns. More research is needed to establish a clear understanding of how the type, dosage, and duration of corticosteroid therapy influence the onset and severity of mental health disorders. Furthermore, existing research tends to focus on well-known psychiatric conditions, potentially overlooking the nuanced effects corticosteroids may have on less commonly studied disorders. To ensure the safe use of corticosteroids, pharmacists and physicians must be aware of their potential psychiatric side effects. This includes proper patient education, monitoring for early signs of mood disturbances, and tailoring corticosteroid treatment plans to individual risk factors. By doing so, healthcare providers can optimize treatment outcomes while minimizing the risk of corticosteroid-induced psychiatric disturbances.

## Data Availability

No new data were created or analyzed in this study. Data sharing is not applicable to this article as it is a narrative review based on previously published studies, which are appropriately cited in the text.

## Supplementary Materials

The following supporting information can be downloaded at: https://www.mdpi.com/article/10.3390/diseases12120300/s1, Figure S1: title PRISMA flow diagram of the review.
