# Supplementary figures and images for: Corticosteroid-Induced Psychiatric Disorders: Mechanisms, Outcomes, and Clinical Implications
Source: Diseases. 2024 Nov 23;12(12):300. doi: 10.3390/diseases12120300 (PMC11675195; doi:10.3390/diseases12120300)

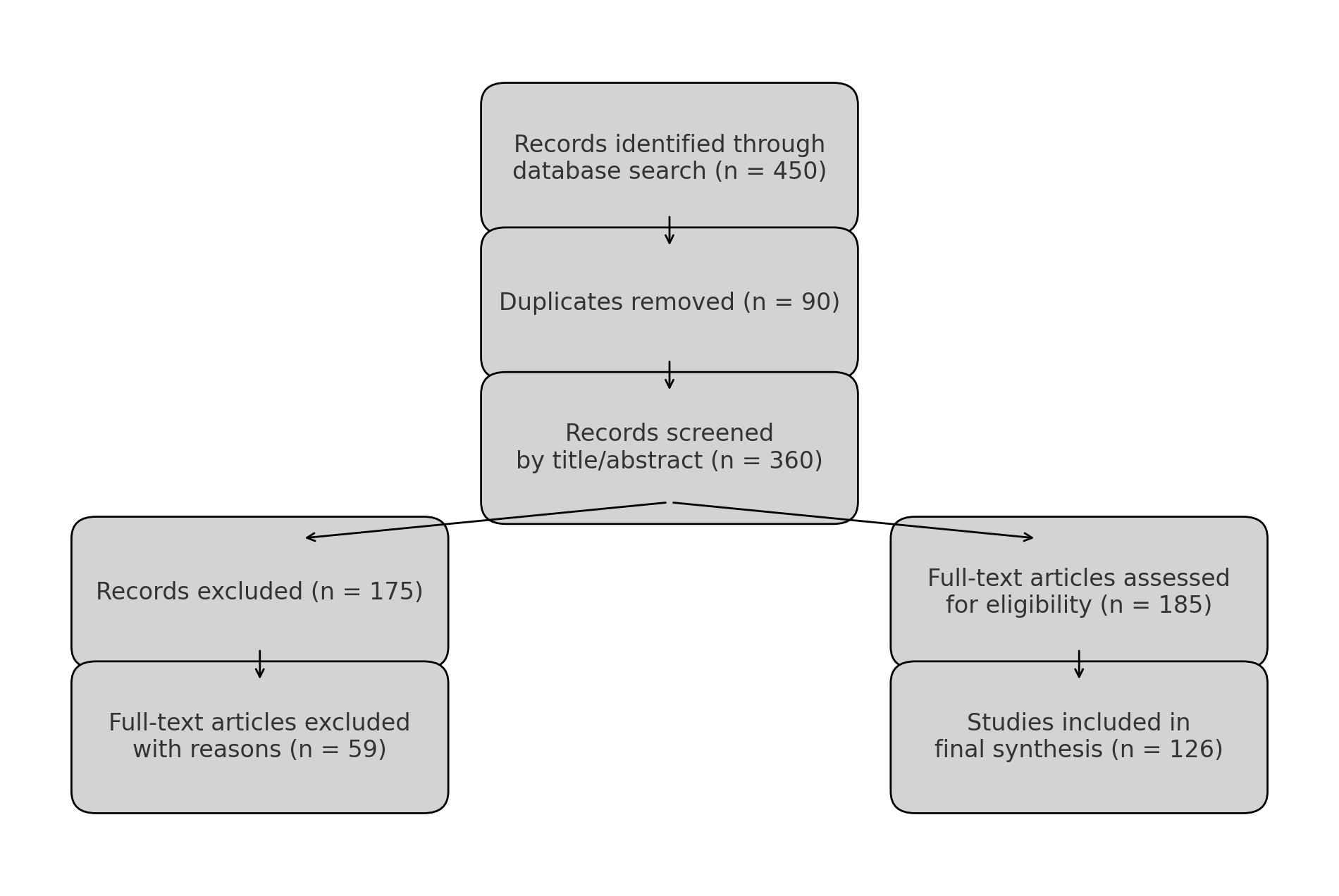

Supplement: Supplementary file 1 [file diseases-12-00300-s001.zip › diseases-3266627-supplementary.png]
